# Spiritual Coping and Duration Since Diagnosis Associated With Quality of Life Among Women With HIV: A Cross‐Sectional Study

**DOI:** 10.1155/arat/8997271

**Published:** 2026-06-30

**Authors:** Rosanti Muchsin, Esti Yunitasari, Abu Bakar, Yulis Hati

**Affiliations:** ^1^ Department of Nursing, Faculty of Nursing, Airlangga University, Kampus C UNAIR Mulyorejo Street, Surabaya, East Java, 60115, Indonesia, unair.ac.id; ^2^ Department of Nursing, Health Science of Faculty, University of Haji North Sumatra, Selamat Lurus Street No. 73 SA Sitirejo III Village Medan Amplas District, Medan, North Sumatra, 20226, Indonesia

**Keywords:** duration since diagnosis, HIV, quality of life, spiritual coping, women

## Abstract

**Background:**

Women with HIV (WWHIV) are a vulnerable group that faces medical challenges as well as psychological, social, and spiritual pressures that affect quality of life. Improving their quality of life requires a holistic approach that considers nonmedical factors alongside social and cultural ones. This study aims to evaluate the association between spiritual coping, duration since diagnosis, and quality of life among WWHIV.

**Methods:**

This study employed a cross‐sectional design and was conducted at a government hospital from March 2024 to August 2024. A total of 120 WWHIV were recruited through purposive sampling based on predefined inclusion and exclusion criteria. Quality of life was assessed with an adapted instrument based on the WHOQOL framework, and spiritual coping was measured with a modified spiritual coping scale. Data were analyzed using Spearman’s rank correlation and ordinal logistic regression.

**Results:**

Spiritual coping was positively related to quality of life (*r* = 0.560, *p* < 0.001). The duration since diagnosis was also positively associated with quality of life (*r* = 0.402, *p* < 0.001). Ordinal logistic regression showed that low spiritual coping was associated with lower odds of a higher quality of life (OR = 0.014, 95% CI = 0.002–0.096, *p* < 0.001). Participants diagnosed for < 6 months and 7–12 months also had lower odds of higher quality of life than those diagnosed for more than 2 years (OR = 0.157, 95% CI = 0.034–0.727 and OR = 0.067, 95% CI = 0.011–0.425, with *p* < 0.05). The model explained 47.5%–55.8% of the variance in quality of life (Nagelkerke *R* ^2^ = 0.558).

**Conclusion:**

Spiritual coping and duration since diagnosis were significantly associated with quality of life among WWHIV. These findings highlight the importance of integrating psychosocial and spiritual support into HIV care, particularly during the early postdiagnosis period.

## 1. Introduction

Quality of life is an essential indicator in the success of long‐term management of HIV/AIDS, especially in the midst of the paradigm shift of HIV from a deadly disease to a chronic condition that can be controlled through antiretroviral therapy [1, 2]. Various factors are known to contribute to the quality of life of people with HIV, ranging from clinical to psychosocial aspects [3, 4]. Conceptually, quality of life is a multidimensional construct that encompasses physical, psychological, social, and environmental domains [5]. Building on this perspective, Basavaraj et al. [6] and other studies [7] have emphasized that, in the context of chronic conditions such as HIV, independence and spirituality are also important components for a more comprehensive understanding of quality of life. This perspective supports a broader conceptualization of quality of life that integrates both functional independence and spiritual dimensions.

In this context, women with HIV (WWHIV) are a very vulnerable group because they face the double burden of medical challenges and psychosocial and spiritual pressures, which may affect their quality of life [8, 9].

This study examined the association between selected psychosocial and clinical factors and the quality of life of WWHIV. Two main variables were chosen as the focus of the study, namely spiritual coping and length of diagnosis. The selection of these two variables is based on theoretical and empirical considerations. Spiritual coping is a form of adaptation mechanism that has been shown to help individuals manage stress and anxiety, and improve the meaning of life, especially in a religious cultural context [10–12]. Spiritual coping can be understood as a cognitive and emotional regulatory mechanism that enables individuals to interpret stressful experiences through a transcendent meaning system [11].

Based on religious coping theory developed by Kenneth I. Pargament, individuals who utilize spiritual resources tend to demonstrate better stress management, enhanced meaning‐making, and improved psychological stability when facing chronic illness such as HIV [11, 13]. In this context, spiritual coping functions as a psychosocial factor associated with quality of life [14].

Spiritual coping can be done actively through prayer, worship, or meditation or passively by surrendering to God’s will. For WWHIV, this strategy has been shown to provide inner peace, strengthen life expectancy, and help deal with the stigma and social pressures they face. Research shows that increased spiritual coping is associated with decreased anxiety and depression, improved medication adherence, increased CD4 counts, and improved immune‐related outcomes [15, 16]. In addition, higher levels of spiritual coping are also linked to improved overall quality of life [17, 18].

The duration since diagnosis is considered an important factor, as a longer duration may facilitate psychological adaptation. A longer duration since diagnosis may allow individuals to develop acceptance gradually, adjust coping strategies, and strengthen social and psychological resilience [19, 20]. In addition to theoretical perspectives on adaptation, recent studies suggest that a longer duration since HIV diagnosis is associated with improved adjustment, treatment engagement, and better quality of life outcomes [21].

Therefore, spiritual coping and the duration since diagnosis can be conceptualized as interrelated psychosocial processes that jointly shape quality of life. Spiritual coping represents an internal mechanism of emotional regulation and meaning‐making, while duration since diagnosis reflects a temporal process of psychological adaptation. Together, these two factors form a conceptual framework for understanding variations in quality of life among WWHIV.

Most previous studies have tested only one of the two variables separately and have not specifically highlighted the female population. This study integrates both variables within a single analytical framework and specifically targets groups of WWHIV, who are often marginalized in HIV/AIDS studies. Spiritual coping research has rarely quantitatively examined the mechanisms underlying its association with overall quality‐of‐life dimensions, particularly with respect to the length of diagnosis [22, 23]. In addition, empirical data on the most effective coping strategies used by WWHIV to address health and social challenges are also limited. These relationships suggest that spiritual coping and duration since diagnosis may function as key psychosocial factors associated with quality of life, providing a conceptual basis for examining their roles within a single analytical framework. Therefore, this study aims to examine the association between spiritual coping, duration since diagnosis, and quality of life among WWHIV using a cross‐sectional design.

## 2. Materials and Methods

### 2.1. Study Design

This cross‐sectional study was conducted from March to August 2024.

### 2.2. Participants and Study Site

The study population consisted of 483 WWHIV who were registered at a specialized HIV care clinic in a government hospital. A total of 120 participants were recruited using purposive sampling based on predefined inclusion and exclusion criteria relevant to the study objectives. Participants were consecutively recruited during routine visits to the HIV clinic throughout the data collection period. All female patients who met the eligibility criteria and attended the clinic during the study period were invited to participate. Potential participants were identified from medical records and screened by the research team with the help of healthcare workers according to the inclusion and exclusion criteria.

Eligible individuals were approached directly, informed about the study’s objectives and procedures, and invited to participate voluntarily. Those who declined were not enrolled in the study. The most common reason for refusal was concern regarding privacy and confidentiality. Written informed consent was obtained from all participants before data collection.

The inclusion criteria were (1) WWHIV between the ages of 20 and 49; (2) diagnosed with HIV at clinical stage 1 or 2, as confirmed by a physician; (3) able to communicate effectively; and (4) willing to participate in the study. The exclusion criterion was being pregnant, as confirmed by self‐report and the attending healthcare provider.

Sample size adequacy was assessed based on commonly cited multivariate analysis guidelines, which suggest a minimum of 10–20 observations per predictor variable [24]. More recent methodological literature emphasizes that such rules should be applied cautiously, as model performance is influenced by factors such as sampling approach, data characteristics, and model complexity [25]. In this study, a sample size of 120 participants was considered adequate for ordinal logistic regression, given the inclusion of two main predictor variables.

### 2.3. Measurement Instruments

Socio‐demographic variables were collected using a standardized questionnaire developed by the researchers. The questionnaire asked about age, marital status, educational level, occupation, and how long participants had been diagnosed with HIV. These variables were used to characterize participants’ backgrounds and investigate potential correlations with quality of life.

In this study, the independent variables were duration since HIV diagnosis and spiritual coping, and the dependent variable was quality of life. Duration since HIV diagnosis was categorized to reflect different phases of adjustment and adaptation following diagnosis, particularly the difference between the early postdiagnosis period and the long‐term experience of living with HIV.

The study used several questionnaires, namely,

1. The quality of life questionnaire.
 Quality of life was assessed using a questionnaire adapted from the WHOQOL framework and informed by the conceptual model proposed by Basavaraj et al. [6]. The instrument was designed to capture key quality‐of‐life dimensions relevant to WWHIV, including physical health, psychological well‐being, independence, social relationships, and spirituality. The instrument was slightly adapted to ensure cultural relevance and suitability for the study population’s characteristics. This process included refining item wording and selecting domains aligned with the study objectives. The environmental domain was not included, as aspects related to daily functioning and autonomy were considered to be represented within the level of independence domain. Additionally, spiritual aspects were considered an important and distinct dimension in this study.
 The quality‐of‐life questionnaire used consists of 21 questions divided into five parts. The first section focuses on assessing physical abilities (4 questions: pain and discomfort, energy and fatigue, and sleep and rest). The second part, the psychology domain, consisted of items assessing emotional well‐being, cognitive function, self‐esteem, body image, and negative emotions. The third part: level of independence (3 questions: mobility, daily life activities, and work capacity). The fourth section: social relationships (five questions: personal relationships, social support, sexual activity, and social engagement). The fifth section investigates spirituality (four questions: calmness and peace, and mental and emotional well‐being). All items were rated on a five‐point Likert scale, with response options adapted to the nature of each item. Higher scores indicated better quality of life.
2. Spiritual coping questionnaire.
 Spiritual coping was measured using *The Scale of Spiritual Faith,* developed by Pargament, Feuille, and Burdzy, and subsequently refined by Alfakseir and Coleman [21]. In this study, the instrument underwent limited adaptation to align with the cultural context and characteristics of WWHIV. The modifications included reducing the number of items from the original Version to 6 and refining the wording of items to improve clarity, cultural relevance, and comprehensibility. These modifications were undertaken without altering the instrument’s core conceptual dimensions, which include accepting God’s decrees and trusting in God’s help. Each question was rated on a five‐point Likert scale ranging from *strongly disagree* to *strongly agree*. Higher scores indicate higher levels of spiritual coping.

### 2.4. Validity and Reliability

Before the main study, the validity and reliability of the instruments were reassessed following adaptations and modifications to the original instruments, using a pilot test with 26 respondents with characteristics similar to those of the study population. Construct validity was tested using Pearson’s product‐moment correlation to examine the relationship between each item’s score and the total score.

The results showed that the quality‐of‐life questionnaire had correlation coefficients ranging from 0.431 to 0.643, while the spiritual coping questionnaire had correlation coefficients ranging from 0.680 to 0.954. All items had calculated *r* values greater than the table *r* value (*r* = 0.374), indicating acceptable item validity. Reliability analysis indicated that the quality‐of‐life questionnaire had a Cronbach’s alpha of 0.675, indicating modest internal consistency. In contrast, the spiritual coping questionnaire demonstrated very good reliability, with a Cronbach’s alpha of 0.922.

The relatively lower reliability score of the quality‐of‐life instrument may be related to the reduction in the number of items during the adaptation process, which can affect inter‐item consistency. Although confirmatory factor analysis was not performed, the adaptation process was guided by established theoretical frameworks to support content relevance and domain consistency.

### 2.5. Data Collection Procedure

Participants who met the eligibility criteria were invited to participate and provided written informed consent before data collection began. The self‐administered questionnaires were completed anonymously in a private area of the hospital waiting room to help maintain confidentiality and minimize social desirability bias. The researcher was available to provide clarification or assistance during questionnaire completion, while ensuring that participants completed the questionnaires independently and without coercion.

Participants were informed that their participation was voluntary, that they could withdraw at any time, and that all information would be kept confidential. After completion, the researcher reviewed the questionnaires for completeness and consistency to ensure data quality before further processing.

Participants received compensation in the form of transportation reimbursement and a small stipend as a token of appreciation for their time and participation. The amount was modest, aligned with local standards, and carefully determined to avoid influencing participants’ decisions to take part in the study.

This study has obtained ethical approval from the Health Research Ethics Committee of University of “Airlangga” under number 3069‐KEPK dated January 22, 2024. This ethical approval aims to ensure that all procedures comply with ethical standards for research involving human participants.

### 2.6. Data Analysis

The collected data were analyzed using the IBM SPSS Statistics program Version 25. Descriptive statistical analysis was used to summarize participants’ characteristics, and results are presented as frequencies, percentages, medians, and interquartile ranges, depending on the data type.

Before conducting the inferential analysis, the data distribution was assessed to determine which statistical tests to use. As the data were not normally distributed and involved ordinal variables, Spearman’s rank correlation was used to examine associations.

Ordinal logistic regression analysis was conducted to examine factors associated with quality of life, given the ordinal nature of the dependent variable. The model included socio‐demographic covariates (age, education, and marital status) to account for potential confounding effects.

The proportional odds assumption was assessed using the parallel lines test, with a nonsignificant result (*p* > 0.05) indicating that the assumption was met. Model adequacy was further evaluated using the goodness‐of‐fit test and pseudo R‐square measures. Statistical significance was set at *p* < 0.05.

## 3. Results

### 3.1. Demographic Characteristics

Table 1 shows that the majority of participants were aged 36–49 years (56.7%), had completed high school (58.3%), and were married (60.8%). Most participants reported sexual transmission as the primary route of HIV infection (90.0%), and 63.3% had been diagnosed with HIV for more than 2 years. Regarding spiritual coping, more than half of the participants (55.0%) demonstrated a high level. Regarding quality of life, the majority of participants were categorized as having a moderate level of overall quality of life (50.8%), and the median total quality‐of‐life score was 74.00 (IQR = 25.00), indicating a generally moderate level of quality of life. The distribution of quality of life across individual domains is presented in Table 2.

**TABLE 1:** Sociodemographic characteristics, spiritual coping, and quality of life of WWHIV (*n* = 120).

Variable	Category	*n* (%)	Median (min–max)	IQR
Age (years)	20–25	7 (5.8)	37.00 (20.00–49.00)	12.00
	26–35	45 (37.5)		
	36–49	68 (56.7)		

Education	No formal education	1 (0.8)	—	—
	Primary school	5 (4.2)		
	Junior high school	14 (11.7)		
	Senior high school	70 (58.3)		
	Diploma	9 (7.5)		
	Bachelor’s degree	19 (15.8)		
	Master’s degree	2 (1.7)		

Marital status	Unmarried	9 (7.5)	—	—
	Married	73 (60.8)		
	Living apart	1 (0.8)		
	Widowed	11 (9.2)		
	Divorce	26 (21.7)		

Transmission history	Syringe	6 (5.0)	—	—
	Sexual	108 (90.0)		
	Blood transfusions	6 (5.0)		

Duration since diagnosis	≤ 6 months	20 (16.7)	4.00 (1.00–4.00)	2.00
	7–12 months	11 (9.2)		
	1–2 years	13 (10.8)		
	> 2 years	76 (63.3)		

Spiritual coping	Low	14 (11.7)	24.00 (6.00–30.00)	8.00
	Moderate	40 (33.3)		
	High	66 (55.0)		

Total quality of life	Low	13 (10.8)	74.00 (29.00–105.00)	25.00
	Moderate	61 (50.8)		
	High	46 (38.3)		

*Note: n* = number of respondents; % = percentage; IQR = interquartile range.

**TABLE 2:** Distribution of quality of life domains among WWHIV (*n* = 120).

Domain	Category	*n* (%)	Median (min–max)	IQR
Physical	Low	14 (11.7)	15.00 (5.00–20.00)	6.00
	Moderate	43 (35.8)		
	High	63 (52.5)		

Psychological	Low	16 (13.3)	18.00 (7.00–25.00)	6.00
	Moderate	47 (39.2)		
	High	57 (47.5)		

Level of independence	Low	12 (10.0)	17.00 (6.00–25.00)	6.00
	Moderate	54 (45.0)		
	High	54 (45.0)		

Social relationships	Low	19 (15.8)	13.00 (4.00–20.00)	6.00
	Moderate	62 (51.7)		
	High	39 (32.5)		

Spirituality	Low	26 (21.7)	13.00 (5.00–20.00)	5.00
	Moderate	53 (44.2)		
	High	41 (34.1)		

Table 2 shows that overall, most participants reported moderate to high levels across all domains. The physical domain showed the highest proportion of participants in the high category (52.5%), followed by the psychological domain (47.5%). In contrast, the social relationships and spirituality domains were predominantly in the moderate category (51.7% and 44.2%, respectively), indicating that these aspects of well‐being were less optimal than other domains.

### 3.2. Correlation Between Independent Variables and Quality of Life

Spearman’s rank correlation analysis was conducted to examine the association between independent variables (duration since diagnosis and spiritual coping) and quality of life among WWHIV. The results are presented in Table 3.

**TABLE 3:** Spearman correlation between independent variables and quality of life.

Independent variables	Spearman’s *ρ*	*p*‐value
Spiritual coping	0.560	< 0.001^∗^
Duration since diagnosis	0.402	< 0.001^∗^

^∗^ *p*‐value < 0.05.

A statistically significant positive correlation was found between spiritual coping and quality of life (*ρ* = 0.560, *p* < 0.001). Duration since diagnosis was also positively correlated with quality of life (*ρ* = 0.402; *p* < 0.001).

### 3.3. Analysis of Ordinal Logistic Regression

The proportional odds assumption was evaluated using the parallel lines test, which showed no statistical significance (*χ* ^2^ = 12.777, df = 16, *p* = 0.689). This indicates that the assumption was met. Therefore, ordinal logistic regression analysis was considered the most appropriate method for examining the relationship between spiritual coping, duration since diagnosis, and quality of life. The results of the ordinal logistic regression analysis are as follows (Table 4).

**TABLE 4:** Ordinal logistic regression analysis of factors associated with quality of life.

Variable	*β*	SE	OR (*e* ^β^)	95% CI	*p* *-*value
Spiritual coping					
Low vs. high	−4.298	0.998	0.014	0.002–0.096	< 0.001^∗^
Moderate vs. high	−0.211	0.481	0.810	0.315–2.077	0.660
Duration since diagnosis					
≤ 6 months vs. > 2 years	−1.852	0.782	0.157	0.034–0.727	0.018^∗^
7–12 months vs. > 2 years	−2.700	0.941	0.067	0.011–0.425	0.004^∗^
1–2 years vs. > 2 years	0.265	0.698	1.303	0.331–5.118	0.705
Age (continuous)	0.026	0.036	1.026	0.957–1.098	0.469
Marital status					
Unmarried	0.205	1.103	1.227	0.142–10.650	0.853
Married	0.235	0.524	1.265	0.453–3.530	0.654
Living apart	−2.292	2.777	0.101	0.001–23.600	0.409
Divorced	−1.859	0.964	0.155	0.024–1.031	0.054
Education					
No formal education	−3.922	3.379	0.020	0.000–13.900	0.246
Primary school	−0.564	2.128	0.569	0.009–36.880	0.791
Junior high school	−2.817	2.032	0.060	0.001–3.210	0.166
Senior high school	−1.425	1.880	0.240	0.006–9.580	0.449
Diploma	−1.045	2.010	0.352	0.007–18.100	0.603
Bachelor’s degree	0.286	1.935	1.331	0.028–59.100	0.882

*Note:* Reference categories were high spiritual coping for spiritual coping, > 2 years for duration since diagnosis, widowed for marital status, and master’s degree for education level.

Abbreviations: CI = confidence interval, OR = odds ratio, SE = standard error.

^∗^ *p*‐value < 0.05.

An ordinal logistic regression analysis including sociodemographic covariates (age, education, and marital status), as well as spiritual coping and duration since diagnosis. The results showed that participants with low spiritual coping had substantially lower odds of reporting a higher quality of life than those with high spiritual coping (OR = 0.014; 95% CI = 0.002–0.096; *p* < 0.001), indicating a markedly strong association.

In addition, participants who had been diagnosed for less than 6 months and 7–12 months had notably lower odds of higher quality of life than those diagnosed for more than 2 years (OR = 0.157; 95% CI = 0.034–0.727; *p* = 0.018; OR = 0.067; 95% CI = 0.011–0.425; *p* = 0.004), suggesting that individuals in the early stages of diagnosis are considerably more vulnerable to poorer quality of life.

In contrast, moderate levels of spiritual coping and a diagnosis duration of 1–2 years were not significantly associated with quality of life (*p* > 0.05). In the adjusted model, age, education level, and marital status were not significantly associated with quality of life.

### 3.4. Model Fit

The results of the ordinal logistic regression model are presented in Table 5.

**TABLE 5:** Model fit statistics for ordinal logistic regression.

Test	*χ* ^2^	df	*p*‐value
Model fitting	77.35	16	*p* < 0.001^∗^
Goodness of fit (Pearson)	154.87	194	0.982^∗∗^
Goodness of fit (deviation)	130.59	194	1.000^∗∗^
Cox and Snell *R* ^2^	0.475		
Nagelkerke *R* ^2^	0.558		

^∗^ *p* < 0.05 indicates statistical significance.

^∗∗^ *p* > 0.05 in the goodness of fit test indicates that the model fits the data adequately.

The ordinal logistic regression model significantly improved upon the intercept‐only model (*χ* ^2^ = 77.35, df = 16, *p* < 0.001), suggesting that the independent variables collectively explained the variation in quality of life among WWHIV.

Goodness‐of‐fit tests showed that the model fit the data adequately. The Pearson test was not significant (*χ* ^2^ = 154.87, df = 194, *p* = 0.982), and the deviance test also showed a nonsignificant result (*χ* ^2^ = 130.59, df = 194, *p* = 1.000). These results suggest that there is no substantial difference between the observed and expected values. Pseudo R‐square values showed that the model explained 47.5%–55.8% of the variation in quality of life.

## 4. Discussion

The findings of this study showed that spiritual coping and duration of HIV diagnosis were significantly correlated with the quality of life of WWHIV. In particular, a lower level of spiritual coping and a shorter duration since diagnosis (< 1 year) were associated with lower odds of having a higher quality of life.

The regression model demonstrated an adequate fit for the observed data, as indicated by nonsignificant goodness‐of‐fit tests. These results suggest that the identified relationships between the independent variables and quality of life are statistically reliable. The moderate‐to‐high pseudoR‐square values further indicate that the selected variables explain a substantial proportion of the variation in quality of life, supporting the multidimensional nature of quality of life among WWHIV.

The inclusion of socio‐demographic variables in the regression model did not substantially alter the main findings. Spiritual coping and duration since diagnosis remained the factors most consistently associated with quality of life in this study, suggesting that these variables may have a stronger relationship with quality of life compared to the socio‐demographic characteristics included in the model.

### 4.1. The Association Between Spiritual Coping and Quality of Life Among WWHIV

The present study found that higher levels of spiritual coping were associated with better quality of life among WWHIV. Participants with a low level of spiritual coping were more likely to report a poorer quality of life compared to those with high spiritual coping. These findings indicate a significant relationship between spiritual coping and quality of life among WWHIV. However, given the cross‐sectional nature of this study, causal relationships cannot be established. It is possible that stronger spiritual coping contributes to a better quality of life, while, conversely, individuals with a better quality of life may also be more likely to engage in positive spiritual coping practices.

These findings are in line with the theory of religious coping developed by Pargament [26], which states that spirituality‐based coping strategies enable individuals to find meaning, hope, and develop inner strength when facing severe life stressors, including chronic illnesses such as HIV/AIDS.

Spiritual coping provides individuals with a way to manage stress by fostering transcendental relationships through prayer, surrender, and reflection on spiritual values. For WWHIV, these strategies may function as both a psychological defense and a cognitive‐affective mechanism that helps them accept their condition, reduce feelings of hopelessness, and enhance their sense of social and spiritual connection. Previous research supports these findings, showing that higher levels of spirituality are associated with lower anxiety and depression levels, as well as higher life satisfaction in WWHIV [27, 28]. Beyond these psychological benefits, spiritual coping may also be associated with clinical outcomes. Previous studies have suggested that spiritual coping may contribute to clinical stability through indirect pathways, particularly through better medication adherence and emotional regulation [29]. Additionally, other findings suggest that specific coping styles may mediate the relationship between spiritual coping and adherence behaviors [30].

In a sociocultural context such as Indonesia’s, spiritual coping mechanisms may play a particularly prominent role. Spirituality is often deeply embedded in daily life, social relationships, and cultural values. For WWHIV, spiritual practices may become even more important after receiving an HIV diagnosis. Emotional distress and major life changes following diagnosis may prompt some individuals to reconnect with their religious beliefs and spiritual practices as sources of comfort, meaning, and inner strength. Therefore, spirituality may function both as an individual coping resource and as part of a broader cultural and religious framework that helps WWHIV emotionally adapt to living with HIV.

Although these findings highlight the importance of spirituality, they must be interpreted with consideration for potential variations among individuals and contexts. Not all WWHIV experience or use spiritual coping in the same way, indicating the need for context‐sensitive approaches in future research.

### 4.2. The Association Between Duration Since HIV Diagnosis and Quality of Life Among WWHIV

The findings of this study indicate that the length of time since diagnosis is significantly associated with quality of life. Participants who had been diagnosed for ≤ 6 months and 7–12 months were less likely to have a better quality of life compared to those who had been diagnosed for more than 2 years.

These findings may reflect differences in psychological and social adaptation processes among WWHIV. Individuals who have lived longer with HIV may differ in their coping experiences and engagement with healthcare services.

This interpretation aligns with the stress‐disease adaptation model, which suggests that individuals typically go through several psychological stages, including denial, anger, bargaining, depression, and acceptance [31]. In the context of chronic conditions such as HIV, a longer duration since diagnosis may be associated with gradual adaptation over time. While a longer duration since diagnosis was associated with better quality of life, the temporal direction of this relationship cannot be determined from the present study. Individuals with better psychological adjustment and quality of life may be more likely to remain engaged in healthcare services and, therefore, be included in the study sample. This potential survivor bias may partly explain the observed relationship between longer duration since diagnosis and better quality of life.

Previous studies have reported similar patterns, namely that individuals who have known their HIV status for a longer period tend to report more stable psychological conditions and a better quality of life [32, 33], which may be related to the development of adaptive coping strategies, increased acceptance of the diagnosis, and the integration of treatment into daily life.

These findings suggest that individuals in the early stages after being diagnosed may have difficulty adjusting to major life changes and the uncertainty associated with their condition. At this stage, they may still be coming to terms with their diagnosis and may not have developed effective coping mechanisms or a stable support system yet.

Conversely, WWHIV for a longer period may exhibit different patterns of psychological adjustment, coping strategies, and health service utilization. While the time since diagnosis cannot be changed, these results indicate that the period immediately after diagnosis is a critical time to provide psychosocial and clinical support to maintain or improve quality of life.

In this study, age was not significantly associated with quality of life in the regression model, suggesting that its relationship with quality of life may be indirect or mediated by other psychosocial or contextual factors. Similarly, socio‐demographic variables such as education and marital status were not significantly associated with quality of life, which may indicate that their relationships with quality of life are context‐dependent or related to other unmeasured factors.

## 5. Conclusions

This study found that spiritual coping and duration since diagnosis were significantly associated with quality of life among WWHIV. Higher levels of spiritual coping and longer duration since diagnosis were associated with better quality of life.

These findings highlight the importance of integrating spiritual support and psychosocial care into HIV services, particularly during the early postdiagnosis period when individuals may still be adjusting to living with HIV.

This study also contributes to the literature by providing a context‐specific understanding of how spiritual coping operates within a culturally embedded setting, where religious values play an important role in shaping health‐related quality of life.

Future research using longitudinal designs and interventional approaches, as well as broader psychosocial and clinical variables, is recommended to further examine the mechanisms underlying quality of life among WWHIV.

## 6. Limitations

This study has several limitations. The cross‐sectional design precludes causal inferences, and the observed relationships between spiritual coping, duration since diagnosis, and quality of life should be interpreted as associations. Using purposive sampling and recruiting from a single specialized HIV clinic may introduce selection bias and limit the generalizability of the findings. Although participants were recruited consecutively during routine clinic visits, those who declined, especially due to privacy and confidentiality concerns, may differ from those who agreed.

Although key socio‐demographic variables were included in the regression model, residual confounding by unmeasured factors (e.g., mental health status, ART adherence, and social support) cannot be excluded.

In addition, the WHOQOL instruments were adapted from existing frameworks. Although validity and reliability testing were conducted, no confirmatory factor analysis was performed following the adaptation process. Therefore, some limitations regarding construct validity and comparability with other studies should be considered.

Furthermore, the findings should be interpreted within the specific sociocultural context in which the study was conducted, as this may limit their applicability to other populations.

Future studies using longitudinal designs, probability sampling, a broader range of psychosocial variables, and more extensive psychometric evaluation, including confirmatory factor analysis of adapted instruments, are recommended to provide a more comprehensive understanding of quality of life among WWHIV. Future research may also explore interventional approaches, including spiritual support programs and psychosocial resources, to improve the quality of life among WWHIV.

## Funding

The authors received no financial support for the research, authorship, or publication.

## Conflicts of Interest

The authors declare no conflicts of interest.

## Data Availability

All data produced or examined in this investigation are incorporated in this published article. The anonymized raw datasets (Excel and SPSS files) that underpin this study’s findings are securely maintained by the corresponding author and can be accessed upon reasonable request. Owing to ethical and confidentiality concerns regarding WWHIV, the raw data cannot be disclosed to the public.
